# A New Digital Health Program for Patients With Inflammatory Bowel Disease: Preliminary Program Evaluation

**DOI:** 10.2196/39331

**Published:** 2023-04-28

**Authors:** Saemundur Jon Oddsson, Thrudur Gunnarsdottir, Lilja Gudrun Johannsdottir, Maria Lovisa Amundadottir, Arna Frimannsdottir, Pauliina Molander, Anna Karoliina Ylanne, Anna Sigridur Islind, Maria Oskarsdottir, Tryggvi Thorgeirsson

**Affiliations:** 1 Sidekick Health Digital Therapeutics Reykjavik Iceland; 2 Reykjavik University Reykjavik Iceland; 3 Gallup Reykjavik Iceland; 4 Abdominal Center Gastroenterology Helsinki University Hospital Helsinki Finland; 5 University of Helsinki Helsinki Finland; 6 Pfizer Kangasala Finland

**Keywords:** inflammatory bowel disease, self-management, stress, fatigue, sleep, digital platform, digital therapeutics, real-world evidence, mobile phone

## Abstract

**Background:**

Inflammatory bowel disease (IBD) causes chronic inflammation of the gastrointestinal tract. IBD is characterized by an unpredictable disease course that varies greatly between individuals and alternates between the periods of relapse and remission. A low energy level (fatigue) is a common symptom, whereas stress and reduced sleep quality may be the triggering factors. Therapeutic guidelines call for effective disease assessment, early intervention, and personalized care using a treat-to-target approach, which may be difficult to achieve through typical time- and resource-constrained standard care. Providing patients with a digital health program that incorporates helpful self-management features and patient support to complement standard care may be optimal for improving the disease course.

**Objective:**

This study aimed to perform a preliminary program evaluation, analyzing engagement and preliminary effectiveness and the effect on participants’ energy levels (fatigue), stress, and sleep quality, of a newly developed 16-week digital health program (SK-311 and SK-321) for patients with IBD.

**Methods:**

Adults with IBD were recruited to participate in a real-world, live, digital health program via Finnish IBD patient association websites and social media. No inclusion or exclusion criteria were applied for this study. Baseline characteristics were entered by the participants upon sign-up. Platform engagement was measured by tracking the participants’ event logs. The outcome measures of stress, energy levels (fatigue), and quality of sleep were reported by participants through the platform.

**Results:**

Of the 444 adults who registered for the digital health program, 205 (46.2%) were included in the intention-to-treat sample. The intention-to-treat participants logged events on average 41 times per week (5.9 times per day) during the weeks in which they were active on the digital platform. More women than men participated in the intervention (126/205, 88.7%). The mean age of the participants was 40.3 (SD 11.5) years, and their mean BMI was 27.9 (SD 6.0) kg/m^2^. In total, 80 people provided the required outcome measures during weeks 12 to 16 (completers). Treatment completion was strongly predicted by the number of active days in week 1. Analysis of the completers (80/205, 39%) showed significant improvements for stress (*t*_79_=4.57; *P*<.001; percentage change=−23.26%) and energy levels (*t*_79_=−2.44; *P*=.017; percentage change=9.48%); however, no significant improvements were observed for quality of sleep (*t*_79_=−1.32; *P*=.19).

**Conclusions:**

These results support the feasibility of a digital health program for patients with IBD (SK-311 and SK-321) and suggest that treatment completion might have a substantial positive effect on patient-reported stress and energy levels in a real-world setting. These findings are promising and provide initial support for using the Sidekick Health digital health program to supplement standard care for patients with IBD.

## Introduction

### Background

Inflammatory bowel disease (IBD) is a chronic disease often diagnosed early in life, during childhood or early adulthood [1]. The disease causes chronic inflammation of the gastrointestinal tract and clinically includes Crohn disease (CD), ulcerative colitis (UC), and unclassified IBD [2]. The incidence of IBD has been steadily rising worldwide, and it is considered one of the most common gastrointestinal diseases since the beginning of the 21st century [3]. Currently, the prevalence rates are highest in high-income countries (Europe and North America). However, over the last decades, its prevalence has also rapidly increased in low-income countries in Asia, Africa, and South America, emphasizing the increasing global burden of IBD [3,4].

Although the etiology of IBD remains largely unknown, it has been postulated to result from an uncontrolled immune response to a trigger in genetically prone individuals [5]. A trigger may include physiological and environmental factors such as diet, smoking, and stress, although the role of these factors in IBD is not entirely clear [6]. IBD is characterized by an unpredictable disease course that varies greatly between individuals and alternates between periods of active (relapse or flare-ups) and inactive (remission) disease [7]. The main symptoms include episodes of abdominal pain, diarrhea, rectal bleeding, weight loss, bowel urgency, and fatigue or lack of energy [1,5].

In a recent study [8], fatigue was reported as one of the most common disabling symptoms (86.9%) in adult patients with IBD. Furthermore, among patients with active disease, sleep problems, in addition to fatigue, were reported significantly more often than among patients in remission. Notably, stress was perceived by these patients as one of the primary triggers for IBD flare-ups [8], although reduced sleep quality and increased stress may also be a result of increased disease activity, pain, and fatigue during a flare-up [9,10]. Nevertheless, growing evidence suggests that reduced sleep quality, and particularly stress, may act as a promoting factor or relapse-triggering factor for IBD [11,12]. Reduced sleep quality and stress, therefore, seem to both *trigger* fatigue and other IBD symptoms and *result* from IBD, which points to a challenging and negative vicious circle between these interrelated factors.

In general, the unpredictable nature and often debilitating effects of various coexisting IBD symptoms lead patients to consistently report a low quality of life, high rates of health care use, and decreased productivity [13,14]. As there is currently no cure for IBD, treatments are primarily focused on symptom management, mucosal healing, and prevention of relapses to improve patient quality of life and reduce the cost and burden of care. When applying conventional treatment approaches, almost 50% of the patients with CD and 16% of the patients with UC require intestinal surgery within 10 years of diagnosis [15,16]. Strikingly, up to 70% of individuals with CD and approximately 30% of those with UC will undergo gastrointestinal surgery at least once in their lives [17]. Therefore, a treat-to-target (T2T) approach, involving close monitoring of specific and objective measures of inflammation, has recently been advocated for IBD to improve long-term patient outcomes and reduce the need for surgery [18,19]. The T2T approach aims to reduce or prevent disease progression, prevent bowel damage, and promote mucosal healing by encouraging personalized care and early intervention [18,20]. However, T2T approaches are challenging to implement in clinical practice, where patients are only seen every few months for a limited amount of time. Providing patients with effective self-management strategies to complement outpatient care is therefore optimal.

### Digital Health Programs

With the rapid rise of digitalization, digital health programs may be a promising medium for complementing traditional outpatient care and achieving a T2T goal. Digital health programs may facilitate improved communication between patients and health care providers. They may also provide patients with a means of tracking their symptoms and medical information, learning about their condition, and getting their questions answered. A study of the effects of smartphone interventions on long-term health management of chronic diseases found that patients who used digital interventions felt more secure about their condition, were more involved in their own care plans, and felt better taken care of outside the hospital or clinical setting [21]. Furthermore, digital interventions have been associated with decrease in health care costs and increase in convenience, productivity, and efficiency [22].

Studies investigating digital interventions for other chronic diseases such as diabetes, cardiovascular diseases, asthma, and gastrointestinal diseases have shown promising improvements in clinical outcomes in some, although not all, studies [23,24]. Specifically, the digital health programs delivered via the Sidekick Health digital therapeutics platform have previously shown promising results in treating obesity and type 2 diabetes [25,26]. Recent studies have noted that research into platforms for gastrointestinal diseases is limited compared with that for other chronic diseases and that most of the platforms have a lack of professional medical involvement [27]. Consequently, the evidence supporting IBD-specific platforms, particularly those involving medical professionals’ involvement, is limited [28].

### Study Objective

The Sidekick Health digital health program (SK-311 and SK-321) for IBD was developed based on evidence-based clinical guidelines with expert medical and behavioral involvement. The intervention was recently deployed for use among patients with IBD in Finland and launched for the first time in May 2020. This preliminary program evaluation was performed to assess engagement with the digital platform and to investigate the initial outcomes from this newly developed digital health program delivered via the Sidekick Health platform on participants’ energy levels (fatigue), stress, and sleep quality, as all these factors play an important role in the course of IBD.

## Methods

### The Sidekick Health Digital Platform

**Figure 1:**
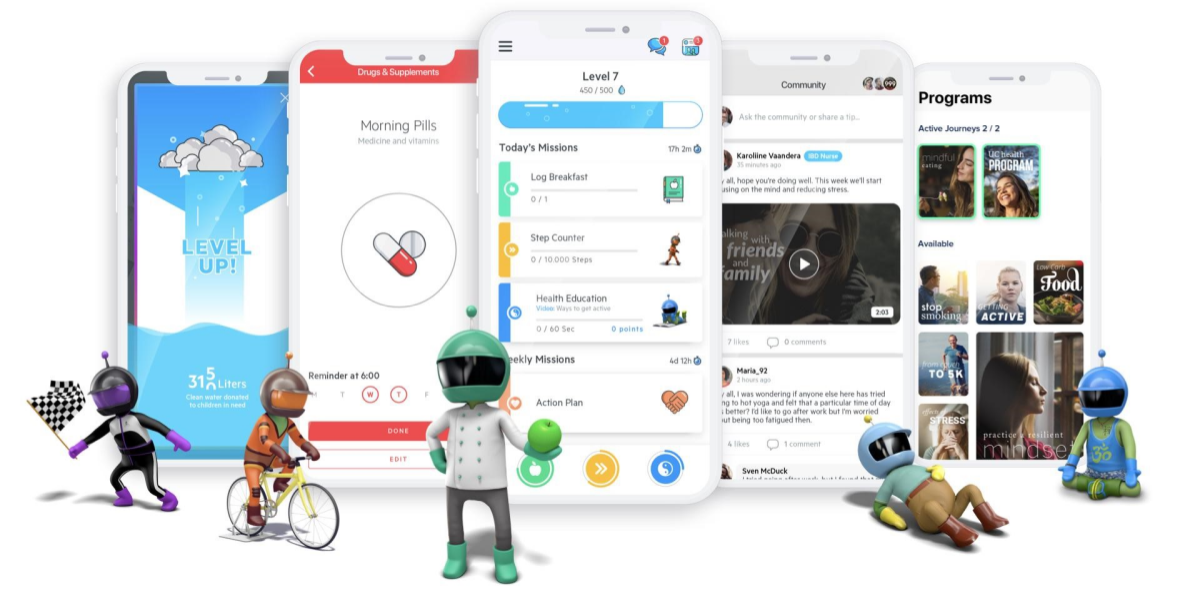
The Sidekick Health digital platform.

Sidekick Health digital therapeutics (Sidekick) is a gamified digital platform that specializes in the delivery of evidence-based digital health programs, including patient support and remote patient monitoring [26]. The platform combines an evidence-based clinical approach with behavioral economic principles and gamification [29]. Sidekick has developed a range of digital health programs for various therapeutic areas, including IBD. These interventions include personalized disease-specific education and treatment support, personalized behavior change goals, action plans, and feedback. Behavioral and medical experts have developed the interventions based on the latest evidence-based clinical guidelines and behavior change theories. The Sidekick platform [30] was designed to increase the frequency of healthy behaviors through goal setting, self-monitoring, and completion of health-related tasks (ie, gamification of tasks) in 4 main categories: nutrition (food), physical activity (move), stress management and psychoeducation (mind), and health (tracking of medications, supplements, and health-related outcomes; Figure 1). By completing gamified tasks, participants accumulate points that provide recognition (eg, in the form of badges) and altruistic rewards (eg, charitable water donations).

### The IBD Digital Health Program (SK-311 and SK-321)

The overarching goal of this newly developed Sidekick Health’s IBD digital health program (SK-311 for patients with UC and SK-321 for patients with CD) is to improve patients’ well-being. Primary targets include reducing stress levels, improving quality of sleep, and improving energy levels. These targets are addressed through learning about disease, learning about cognitive behavioral strategies for behavior change, practicing mindfulness and meditation, increasing physical activity levels, and improving nutrition.

Previous studies have associated lifestyle changes with improved quality of life in patients with IBD [31]. In particular, a growing body of research has shown that patients with IBD might have psychosocial issues, including stress, anxiety, and depression, which can benefit from nonpharmacological interventions [32-34]. Among these, cognitive behavioral therapies, delivered in person or web based [35,36], and mindfulness training [37] have shown encouraging results in improving psychological symptoms and the quality of life of patients with IBD.

The program contained 16 modules in the interventional program. Each module consisted of 1 week of intervention, which included 3-7 psychoeducational videos aimed at teaching or training new skills (skills related, among others, to resilience, self-care, nutritional habits, sleeping, physical activity, and how to change habits). The modules ran in a preset order, but participants could rewatch videos from previous modules in the app library. Each week, the patient was provided with automatic messages and personalized daily missions to complete certain tasks within the platform such as being physically active; practicing meditation and mindfulness; drinking water; eating well; watching psychoeducational videos; and rating their quality of sleep, stress, and energy levels. Patients are also provided with a community feed, where information is provided about general health and IBD-related topics. In addition, a specialized IBD nurse monitors patient activity and sends out weekly feedback and motivational messages. Via the care portal, the nurse can see all the activity users log in the app. On the basis of the activity logged, in combination with the program’s or participants’ personal goals, the nurse provides personalized feedback. This feedback can be positive, negative, or instructional, although nurses are recommended to preferably combine instructional and positive feedback. For instance, in weeks that are focused on nutrition, participants may be asked to take pictures of their food and rate the healthiness and portion size. The nurse can then provide feedback on the food and the rating, perhaps suggesting healthier options or simply congratulating them on the healthy choice they made.

The *minimum* weekly in-app tasks that participants were encouraged to complete were to perform 150 minutes of physical activity; log stress, sleep, and energy levels 3 times per week; eat 2 healthy meals daily; perform 2 guided meditations and 1 mindfulness exercise per week; and walk 21,000 steps per week. The weekly number of steps is automatically personalized; the weekly number is adjusted up (or down) based on the participant’s step count in the previous week (but will not be <3000 steps per day).

### Data Collection

The data reported in this preliminary program evaluation were collected using the Sidekick Health platform in a real-world setting. The platform has restricted access to personal data, including encryption according to the military-grade 256-bit Advanced Encryption Standard, and is compliant with the relevant regulations (eg, General Data Protection Regulations). For this evaluation, anonymized data were extracted for further processing and analysis. No personal or identifiable data were included in the data set, which consisted of participant demographics and platform interactions.

### Patient Recruitment

Potential participants were recruited from Finland via local IBD patient association websites and social media (Facebook and Instagram advertisements). Potential participants could click on posts through the websites and social media, which directed them to a landing page [38]. From there, they received a link with instructions on how to join the intervention. Participants who (1) joined the intervention, (2) provided informed consent, (3) logged at least 1 mission during week 1, and (4) provided the patient-reported outcome metrics of interest for this study during the first week of their intervention (stress, energy levels, and quality of sleep) constituted the intention-to-treat (ITT) sample [39]. Participants who also provided the outcome metrics between weeks 12 and 16 and were active for at least 12 weeks (80/205, 39%) were considered completers.

### Measurements

#### Baseline Characteristics

Participants entered baseline demographic characteristics (date of birth, gender, height, and weight) upon platform sign-up (intervention onboarding). BMI (kg/m^2^) was calculated based on participants’ height and weight, and age was calculated from participants’ date of birth.

#### Engagement

Platform engagement was measured via participants’ event logs (missions completed) within the platform (daily and weekly). A participant was considered active if at least 1 mission was completed during a specified period (either a day or a week). On the basis of the event logs, the average weekly platform interactions and average weekly interactions with mission categories (food, move, mind, and health) were calculated for each participant for their total active weeks. Of note, given the nature of our digital health program, engagement with the platform can be considered as a proxy for healthy behaviors adopted by the users in everyday life. Platform engagement during week 1 was quantified via participants’ number of active days during the first week (range 1-7) and by how many onboarding activities (registering demographic information: age, gender, height, and weight) they performed during that week (ranging from 0 to 4).

#### Stress, Energy Levels, and Quality of Sleep

Patient-reported stress, energy levels, and quality of sleep were assessed using a Likert scale ranging from 0 to 10. Logging of stress, energy levels, and quality of sleep scores was encouraged daily (or at least 2 times per week) as part of the intervention’s prescribed daily missions. The average reported scale score during the first week was used as the week 1 score (first observation), and the average reported scale score for a participant’s last active week in the program was used as their last observation.

### Statistical Analysis

Summary statistics were calculated for demographic and baseline characteristics to describe the overall sample and the subgroups designated as completers and noncompleters. To determine whether there were differences between the groups for these variables at baseline, 2-tailed *t* test and chi-square test were performed. Platform engagement was quantified for week 1 as follows: (1) the number of days active during week 1 and (2) the onboarding score. Platform engagement throughout the intervention was quantified as average weekly platform interactions and average weekly interactions for each category (mind, move, food, and health), including average weekly video lessons watched within the mind category. These variables were estimated by calculating mean and SD values across participants and median values and IQR for a more accurate description of nonnormal distributions.

Concerning the analysis of longitudinal clinically relevant outcomes (quality of sleep and energy and stress levels), only participants who completed the program were included in the analysis. Paired sample 2-tailed *t* tests assessed changes between baseline and program completion in self-reported stress, energy levels, and quality of sleep for the completers. The analyses were performed both with and without imputation using the method of last observation carried forward (LOCF) [40] for the missing data at follow-up (week 16). In addition, Cohen *d* and the percentage change (from baseline to the end of the program) were calculated. The Spearman rank correlation coefficient was used to assess the associations between variables. All statistical analyses were conducted using R (version 4.1.2; R Foundation for Statistical Computing) and SPSS Statistics (version 27; IBM Corp; Build 1.0.0.1508; 64-bit edition). Visual inspection and graphic depiction of data were conducted using Seaborn version 0.10.1 (Python Software Foundation), a Python library for data visualization. The study flowchart was generated using Diagrams version 14.4.7 (JGraph Ltd).

### Ethical Considerations

All participants provided informed consent before inclusion in the study. The platform had restricted access to personal data and was compliant with relevant regulations. For this program evaluation, anonymized data were extracted. The participants were not compensated for their participation in the study. The study was exempt from review by The National Bioethics Committee Board of Iceland (Ministry of Welfare). The study was conducted in accordance with the ethical principles of the Declaration of Helsinki 2008.

## Results

### Overview

**Figure 2:**
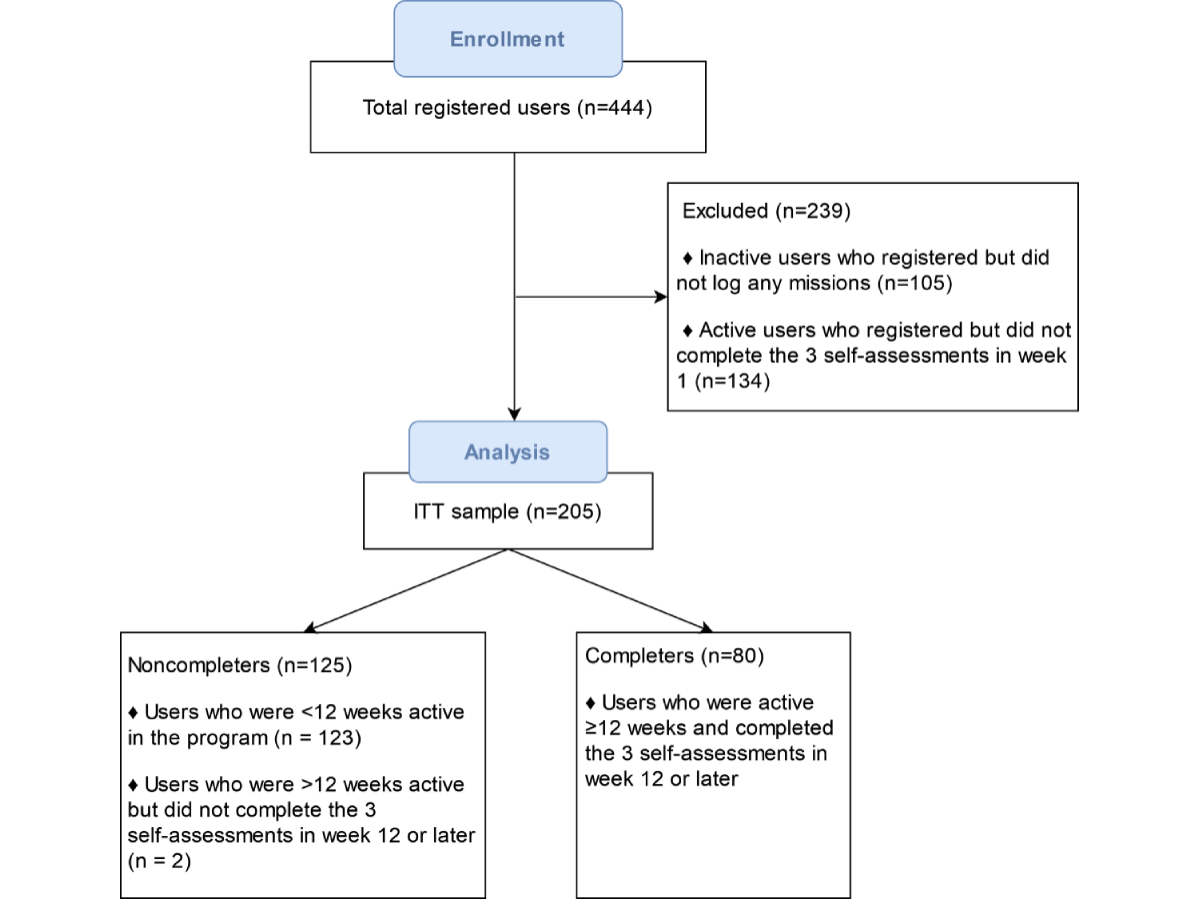
PRISMA (Preferred Reporting Items for Systematic Reviews and Meta-Analyses) flowchart of the study. ITT: intention-to-treat.

The evaluation of the digital health program was launched in Finland on May 21, 2020. Initially, 444 people signed up and provided informed consent. This preliminary program evaluation aimed to examine engagement and retention during the 16 weeks. In addition, the effects of the intervention on 3 clinical outcomes were tested: levels of stress, energy, and quality of sleep. A total of 239 participants who did not report these outcomes or did not log any mission during their first program week were excluded (Figure 2). The derived sample consisted of all potential participants who (1) signed up for the program; (2) provided informed consent; (3) completed the 3 self-assessments in week 1; and (4) had sufficient time from their sign-up date to go through 16 weeks when data were retrieved on February 1, 2021. These criteria resulted in an ITT sample size of 205 participants. Of these 205 participants, 80 (39%) completed the required outcome measures sometime during weeks 12 to 16 and were considered study completers, whereas the other 125 (61%) participants were considered noncompleters. All participants were included in the engagement and retention analyses, whereas only completers were included in the evaluation of clinical outcomes. Figure 2 provides the participant flow through the intervention.

### Participant Characteristics, Engagement, and Well-being at the Start of the Intervention

Baseline characteristics of the total group of participants (ITT sample: 205/205, 100%), completers (80/205, 39%), and noncompleters (125/205, 61%) are provided in Table 1. On average, the participants were aged 40.3 (SD 11.5) years and had a BMI of 27.9 (SD 6.0) kg/m^2^, with 63% (126/200) of participants being overweight or obese. There were significantly more women (126/142, 88.7%) than men (*P*<.05) among those who reported their gender (142/205, 69.3% of the ITT sample). Diagnosis (UC or CD) was not significantly different between the completers and noncompleters.

**Table 1:** Participants’ baseline characteristics, engagement, and well-being (week 1).

					Total intention-to-treat sample (N=205)		Completers (n=80)			Noncompleters (n=125)		*P* value^a^	
**Characteristics**													
	**Female**												.63
		Value, N^a^		142				63	79				
		Value, n (%)		126 (88.7)				55 (87.3)	71 (89.9)				
	**Age (years)**												.44
		Value, N^b^		140				62	78				
		Value, mean (SD)		40.29 (11.52)				39.45 (9.97)	40.96 (12.63)				
	**BMI (kg/m^2^)**												.50
		Value, N^b^		200				78	122				
		Value, mean (SD)		27.85 (6.05)				27.49 (6.22)	28.08 (5.95)				
	**BMI groups, n (%)**												.20
			Underweight (BMI<18.5)	5 (2.4)		4 (5)			1 (0.8)				
			Normal weight (BMI 18.5-24.9)	69 (33.7)		27 (34)			42 (33.6)				
			Overweight (BMI 25-29.9)	64 (31.2)		27 (34)			37 (29.6)				
			Obese (BMI≥30)	62 (30.2)		20 (25)			42 (33.6)				
	**Diagnosis, n (%)**												
			UC^c^	146 (71.2)		62 (78)			84 (67.2)		.11		
			CD^d^	59 (28.8)		18 (23)			41 (32.8)		.11		
**Engagement**													
	**Onboarding score**												.02
		Value, N		205				80	125				
		Value, mean (SD)		3.35 (0.95)				3.54 (0.81)	3.22 (1.01)				
	**Days active week 1**												<.001
		Value, N		205				80	125				
		Value, mean (SD)		4.66 (2.37)				6.63 (0.79)	3.41 (2.19)				
**Well-being,** **mean (SD)**													
			Stress	4.48 (2.23)		4.43 (1.89)			4.52 (2.43)		.76		
			Quality of sleep	6.10 (2.16)		6.16 (1.68)			6.06 (2.42)		.70		
			Energy levels	5.78 (1.79)		5.80 (1.65)			5.77 (1.89)		.89		

^a^Comparison of data from completers and noncompleters.

^b^Number of participants who reported these data.

^c^UC: ulcerative colitis.

^d^CD: Crohn disease.

When looking at engagement during week 1, completers were active (logged events) for more days than noncompleters (mean 6.63, SD 0.79 vs 3.41, SD 2.19; *t*_168.11_=−14.970; *P*<.001). Completers also had a higher onboarding score than noncompleters, indicating that they filled out more background information during the first week (mean 3.54, SD 0.81 vs 3.22, SD 1.01; *t*_192.50_=−2.454; *P*=.02). No other differences were observed between completers and noncompleters at week 1 (Table 1).

Participants’ engagement during their first week of the intervention was determined by counting their number of active days in that week. Of the participants categorized as completers, 74% (59/80) visited the platform every day during their first week. Approximately all participants (77/80, 96%) were active for >4 days during that week. The distribution of active days in week 1 among noncompleters was more diverse than that among completers. Of the 125 noncompleters, 30.4% (n=38) visited the platform only once during their first week, and 70.4% (n=88) were active for <4 days (Multimedia Appendix 1).

### Platform Engagement Throughout the Intervention

We analyzed the number of participants who accessed the platform each week throughout the intervention period (Multimedia Appendix 1). A total of 205 participants joined the intervention during week 1. Of the 205 participants, 141 (68.8%) accessed the platform in week 2 and 103 (50.2%) accessed the platform in week 5. From week 6 onward, most of the remaining participants were retained throughout the intervention.

The weekly number of events logged within the platform, within each platform category (food, move, mind, and health), and data on engagement with the lessons (psychoeducational videos) were tracked (Table 2). On average, participants logged 40.9 (SD 43.7) weekly interactions with the platform (median 26.4), with the most popular category being the food category, where participants logged an average of 18.1 (SD 21.4; median 10.0) interactions.

**Table 2:** Weekly platform engagement.

		Values, mean (SD)	Values, n^a^	Weekly average per participant, median (range; IQR)
**Total interactions**		40.89 (43.69)	205	26.40 (3.00-290.13; 11.94-53.60)
	Food category	18.11 (21.36)	197	10.00 (0.64-120.14; 4.17-24.34)
	Move category	9.36 (12.88)	186	7.42 (0.50-153.88; 2.72-12.22)
	Mind category	11.84 (10.90)	205	8.50 (1.18-72.44; 4.00-16.38)
**Videos watched**		2.63 (1.67)	169	2.63 (0.06-7.67; 1.00-3.81)
	Health category	5.10 (11.07)	127	1.19 (0.06-102.63; 0.31-4.67)

^a^Number of participants that entered data.

### Analysis of Factors Affecting Intervention Completion

Both measures of engagement during week 1, namely, onboarding scores and active days during week 1, differed between completers and noncompleters at baseline (*P*=.02 and *P*<.001; Table 1), and their predictive value for treatment completion was further evaluated using logistic regression models (Table 3). In model A, the variable active days during week 1 was a strong and significant predictor of completion status. However, the variable onboarding score was not a significant predictor of completion status after controlling for participants’ active days during week 1. As shown in model B, the variable active days during week 1 predicted completion status to the same degree as including both variables (active days and onboarding score) in model A (Table 3).

**Table 3:** Logistic regression models for intervention completion.

				OR^a^ (95% CI)		*P* value		Nagelkerke *R*^2^	
**Model A intervention completion**									
	**Predictor variables**								0.606
		Active days in week 1	2.965 (2.145-4.099)		<.001^b^				
		Onboarding score	1.211 (0.773-1.898)		.40				
**Model B intervention completion**									
	**Predictor variables**								0.603
		Active days in week 1	2.981 (2.157-4.120)		<.001				

^a^OR: odds ratio.

^b^The *P*<corrected α is .05/2=.025.

### Analysis of Changes in Quality of Sleep, Stress, and Energy Levels

**Figure 3:**
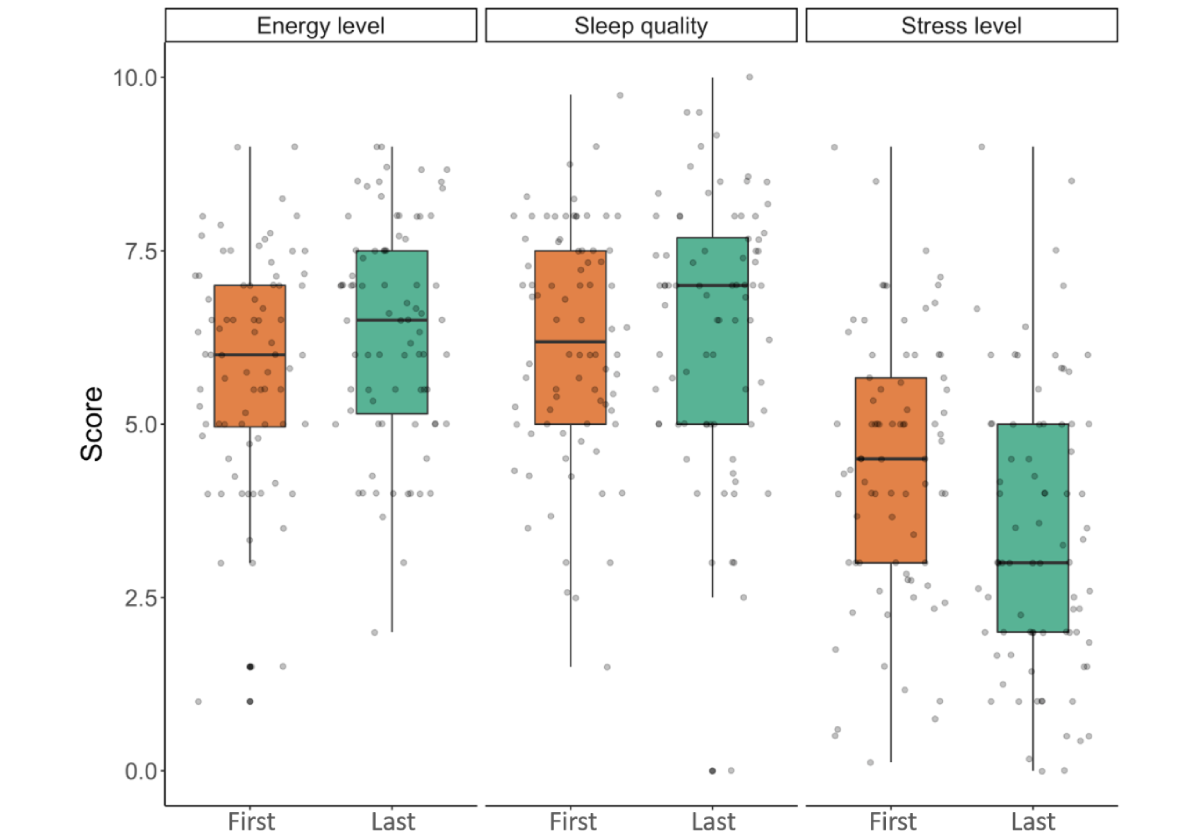
Ratings for energy levels, quality of sleep, and stress levels of completers from first to last observation in the completers group. Energy levels, quality of sleep, and stress levels were scored by participants on a scale of 0 to 10 at the first and last observations. The box plot represents the IQR with Tukey-style whiskers; the horizontal line in the box plot represents the median. Each dot represents the score of a single individual.

The results for changes in quality of sleep, stress, and energy levels for the completers are summarized in Table 4. To exclude the possibility of biased results because of the LOCF imputation method, we performed a paired 2-tailed *t* test analysis both with and without LOCF imputation for missing data. With both approaches, we observed differences in the outcomes for stress, which were significantly reduced (mean difference 1.03, *P*<.001 with LOCF; mean difference 1.16, *P*<.001 without LOCF), and for energy levels, which were significantly increased (mean difference −0.55, *P*=.017 with LOCF; mean difference −0.66, *P*=.01 without LOCF). No significant differences were observed in the outcome sleep quality, either with or without LOCF (Table 4). The distribution of ratings for stress, energy levels, and quality of sleep for the completers is shown in Figure 3.

**Table 4:** Intervention outcomes sleep, stress, and energy in the completers.

			Mean difference (SD; 95% CI)		*t* test (*df*)		*P* value		Change from week 1 to last active week (%)		Cohen *d*
**With LOCF^a^**											
	Sleep	−0.28 (1.90; −0.71 to 0.14)		−1.32 (79)		.19		4.6		−0.147	
	Stress	1.03 (2.02; 0.58 to 1.48)		4.57 (79)		*<.001* ^b^		−23.26		0.511	
	Energy	−0.55 (2.02; −1 to −0.10)		−2.44 (79)		*.017*		9.48		−0.273	
**Without LOCF**											
	Sleep	−0.30 (1.96; −0.78 to 0.18)		−1.27 (68)		.21		4.92		−0.208	
	Stress	1.16 (2.11; 0.65 to 1.67)		4.54 (67)		*<.001*		−26.23		0.541	
	Energy	−0.66 (2.01; −1.16 to −0.17)		−2.67 (65)		*.01*		11.4		−0.34	

^a^LOCF: last observation carried forward.

^b^Italicized *P* values indicate significant differences.

### Associations Between Baseline Characteristics, Engagement Indicators, and Outcomes

For the completers, we also analyzed whether there were any associations between changes in the outcome variables (quality of sleep, stress, and energy levels) and the following variables at baseline: age; BMI; active days in week 1; onboarding score; quality of sleep, stress, and energy levels in week 1; and platform engagement. As shown in Table 5, we observed that for all outcomes, there were significant correlations between Δ changes in the outcome measures and their respective baseline values (sleep *r*=−0.5, stress *r*=0.43, and energy *r*=−0.56). To test whether these correlations indicate a differential effect of the treatment according to the baseline levels, we recomputed them using the Oldham method [41]. The Spearman rank correlation values between Δ changes and their respective baselines with the Oldham method were as follows: sleep *r*=−0.07, stress *r*=−0.13, and energy *r*=−0.008. In addition, Δ for sleep quality correlated with age, stress level at baseline, and the number of food missions.

**Table 5:** Correlation between changes in outcome measures and study variables (n=80).

	Δ^a^ Sleep				Δ Stress				Δ Energy		
	*ρ*	*P* value	Values, n (%)	*ρ*		*P* value	Values, n (%)	*ρ*		*P* value	Values, n (%)
Age	0.27	*.04* ^b^	62 (78)	0		.98	62 (78)	0.11		.39	62 (78)
BMI	−0.21	.06	78 (98)	−0.03		.79	78 (98)	0.03		.81	78 (98)
Active days week 1	0.04	.72	80 (100)	−0.11		.35	80 (100)	0.05		.66	80 (100)
Onboarding score	0	.997	80 (100)	0.04		.74	80 (100)	0.11		.34	80 (100)
Sleep week 1	−0.5	*<.001*	80 (100)	0.11		.34	80 (100)	−0.19		.09	80 (100)
Stress week 1	−0.27	*.02*	80 (100)	0.43		*<.001*	80 (100)	−0.22		.05	80 (100)
Energy week 1	−0.1	.38	80 (100)	0.1		.39	80 (100)	−0.56		*<.001*	80 (100)
Total interactions	0.21	.06	80 (100)	0.06		.58	80 (100)	0.13		.26	80 (100)
Food	0.23	*.04*	80 (100)	0.05		.66	80 (100)	0.08		.50	80 (100)
Move	0.18	.11	80 (100)	0.09		.43	80 (100)	0.18		.11	80 (100)
Mind	0.16	.17	80 (100)	0.05		.64	80 (100)	0.1		.38	80 (100)
Video	0.08	.51	79 (99)	−0.13		.24	79 (99)	0.17		.15	79 (99)
Medical	−0.02	.90	76 (95)	−0.04		.70	76 (95)	0.09		.42	76 (95)

^a^Δ is the difference between the scores of the previous week and week 1.

^b^Italicized *P* values indicate significant differences.

## Discussion

### Principal Findings

In this preliminary program evaluation, we assessed for the first time the real-world benefits of the Sidekick Health’s IBD digital health program. Overall, we observed that participants were highly engaged with the treatment and that 39% (80/205) of the ITT sample completed a minimum of 12 weeks out of the 16-week intervention (completers). Of note, treatment completion was strongly predicted by the number of active days during week 1. In addition, we found that the completers showed statistically significant improvements in stress and energy levels from baseline to treatment completion.

Engagement with the platform is a prerequisite for reaping the benefits of participation. The participants in our sample engaged with the platform on average 41 times per week during the weeks in which they were active, which shows high engagement. Notably, the number of days a participant was active during the first week of the intervention was a robust predictor of intervention completion. Only 9% (7/80) of completers were active for <6 days during the first week. This presumably shows that participants with the highest motivation levels when starting the intervention are, as expected, the most likely to complete the sign-up and onboarding process and then to continue throughout the intervention. Nevertheless, to inform future tailoring of the intervention to individual participants’ characteristics, reasons for participation, and motivation levels, improvements could include baseline measures of these factors to elucidate these findings.

Concerning the clinical outcomes, the findings from this initial evaluation are very promising given the high frequency of experiencing fatigue (low energy levels) by patients with IBD and its debilitating impact on quality of life [8]. In addition, as stress has been documented to be perceived by these patients as one of the leading causes of IBD flare-ups [8], observing reduced stress levels from the first to the last report is very encouraging. As existing IBD treatments are primarily focused on symptom management, prevention of relapses, and a T2T approach, providing patients with effective self-management strategies in the form of a digital platform to complement outpatient care may be optimal. On the basis of the results observed from this initial evaluation of the Sidekick Health real-world, live, digital health program, and consistent with results from other studies [22], this digital health program may be promising as a supplement to standard care. This may facilitate the achievement of T2T goals in patients with IBD.

The observed correlations between each of the outcome changes (sleep, stress, and energy) and their baseline levels could suggest a differential effect of the digital health program according to the starting baseline value (eg, participants with lower energy levels at week 1 showed larger increases in energy levels at the end of the program). However, the negative associations between outcome changes and the corresponding baseline values of the same variables are most likely because of regression to the mean and mathematical coupling [42]. Indeed, when testing these associations using the Oldham method, we could not find any evidence for a differential digital health program effect for different baseline values [41].

### Limitations

This study is a real-world evaluation of an ongoing, live intervention deployed at scale for patients with IBD in Finland. As a result, a limitation of the study is that the initial patient sample may be biased, perhaps leaning toward relatively younger patients, more female patients, and more motivated patients who used to engage with smartphone apps. However, this requires further investigation. Other interindividual differences may also contribute to selection bias; for example, personality traits such as openness and extraversion might be more prevalent among people interested in the new digital platform. In addition, participants with milder symptoms, and therefore higher energy levels, might show a higher adherence to the treatment. However, completers and noncompleters of the treatment did not differ on any of the disease-related variables (energy, sleep, and stress) at baseline. Another limitation of this study may be that the disease status of these patients was not medically ascertained. However, self-reported diagnosis was included, and percentages of UC and CD diagnoses were not found to differ between completers and noncompleters. The sample may also be biased because recruitment occurred through a patient association website and their social marketing efforts that may not have reached all patients with IBD. In addition, for this analysis, we only examined the participants who logged the outcomes of interest during week 1 (baseline). As a result, our sample did not include everyone who registered for the intervention but rather a selected group of participants.

For future evaluations, it would be interesting to examine IBD patient population characteristics in Finland and compare it with the characteristics of the sample that enters our platform. In addition, for our purposes, we will look into our onboarding process and the factors that may affect dropout and engagement in the first days of the intervention, as the data from this study seemed to indicate the importance of high engagement in week 1 for predicting completion. Determining ways to get participants “hooked” on the platform during week 1 might be worth investing in. The collection of additional data on participant characteristics may allow for a better comparison with patients with IBD from the general population, which may help us find more varied methods for participant recruitment. The last notable limitation of this preliminary program evaluation is the lack of validation of the outcome metrics quality of sleep, stress, and energy levels and the absence of a control group for the longitudinal analysis. However, validation of these metrics against clinically validated measures is underway in ongoing research. Moreover, the effect of the digital health program on clinical outcomes in IBD will be tested in a future clinical trial with appropriate controls and randomization, which are beyond the scope of this preliminary program evaluation.

These limitations present an important caveat for generalizing our findings. In addition, the attrition rate of those who entered the intervention was relatively high, although high attrition and differential attrition are common in digital health research [43]. We consider the total retention rate of about 40% during the 16-week program as a fairly good result considering the framework and recruitment. In a recent analysis of retention data from >100,000 participants enrolled in 8 digital health studies, more than half of the participants dropped out after the first week (median retention 5.5 days) but with substantial variability across studies and according to participant characteristics (range 2-26 days) [44]. However, retention in future apps could be further improved by refining the recruitment strategy, by using further tailored in-app rewards, and by adding one-to-one coaching sessions with experts and physicians, thus improving patient monitoring and motivation. The scalability of digital interventions may allow for the recruitment of the sample sizes necessary for adequately powered mediation and moderation tests to explore potential predictors of intervention outcomes further. Of particular interest is the addition of more metrics (eg, disease state, flare-up or remission, pain perception, and symptom severity) to this platform, which will allow for more personalization of the intervention. As relapses in IBD are difficult to predict [7], ideally, adding more features for improving disease monitoring and improving the data collection process will allow us to gather data over time to identify variables that may predict relapse. This information could subsequently improve our interventions further and help prevent flare-ups by early symptom detection and treatment. This would help in maintaining remission and avoiding irreversible bowel damage and decreased quality of life owing to the long-term effects of IBD.

### Conclusions

To conclude, this initial evaluation shows the feasibility and the potential benefits of a real-world digital health program for patients with IBD. Indeed, high engagement and retention with the digital platform seem to positively influence the patient-reported ratings of stress and energy levels. These findings are promising and provide initial support for the use of the digital health program for supplementing traditional outpatient care in the management of IBD.

## Appendix

Multimedia Appendix 1 Participation during the first week and overall during the intervention. (A) Number of active participants during the first week of intervention as a function of the number of active days. The distribution of the intention-to-treat (ITT) sample was split between completers and noncompleters, and 39% (80/205) of the participants were considered completers. (B) Number of active participants throughout the 16-week intervention (ie, participants performing at least 1 activity on the platform during a particular week). The percentage displayed at the top of each bar was calculated based on active participants versus the total ITT sample. Overall, 40% (82/205) of the participants were active during the 12th week of the intervention, and their participation was stable from that week onward.

## Abbreviations

CD: Crohn disease
IBD: inflammatory bowel disease
ITT: intention-to-treat
LOCF: last observation carried forward
T2T: treat-to-target
UC: ulcerative colitis

## Data Availability

The data sets generated or analyzed during this study are available from the corresponding author upon reasonable request.
