# Supplementary figures and images for: A New Digital Health Program for Patients With Inflammatory Bowel Disease: Preliminary Program Evaluation
Source: JMIR Form Res. 2023 Apr 28;7:e39331. doi: 10.2196/39331 (PMC10182451; doi:10.2196/39331)

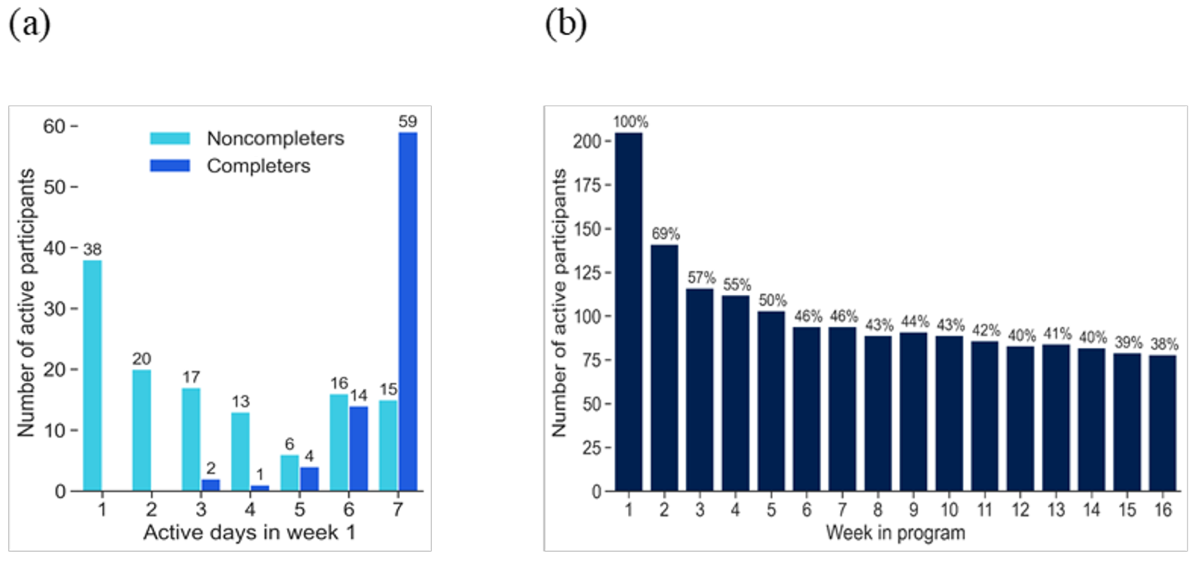

Supplement: Multimedia Appendix 1 [file formative_v7i1e39331_app1.png]
